# Supplementary material for: Association between oral microbiome and seven types of cancers in East Asian population: a two-sample Mendelian randomization analysis
Source: Front Mol Biosci. 2023 Nov 21;10:1327893. doi: 10.3389/fmolb.2023.1327893 (PMC10702768; doi:10.3389/fmolb.2023.1327893)
Supplement: Supplementary file 1 [file DataSheet1.ZIP › Supplementary Materials/MR plots of saliva/saliva═╝/Pancreatic cancer/pheno.3201_to_pancreatic cancer_forest.pdf]

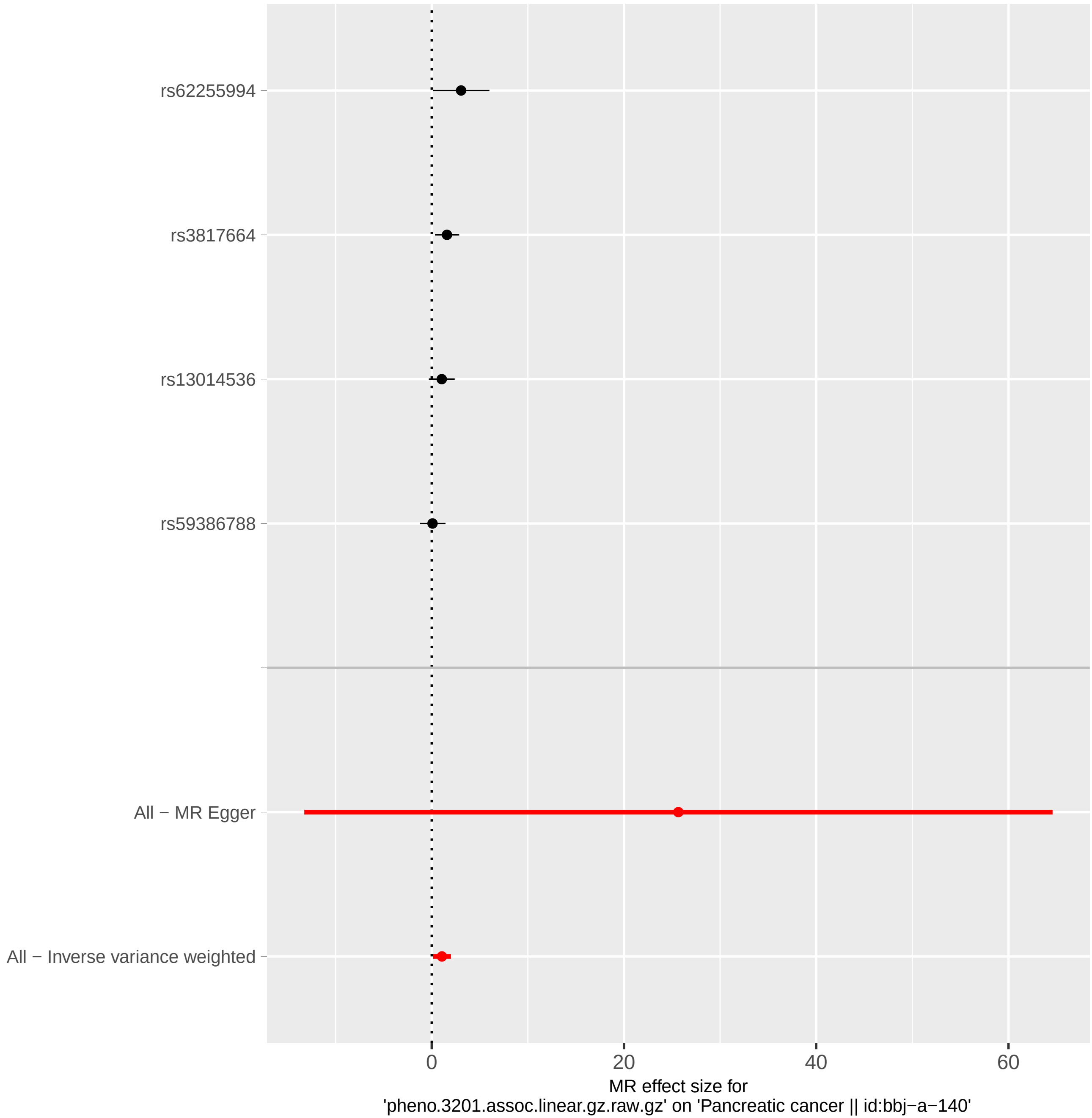

rs62255994
rs3817664
rs13014536
rs59386788
All − MR Egger
All − Inverse variance weighted
0
20
40
60
MR effect size for
'pheno.3201.assoc.linear.gz.raw.gz' on 'Pancreatic cancer || id:bbj−a−140'
